# Limitations of Viscoelastic Constitutive Models for Carbon-Black Reinforced Rubber in Medium Dynamic Strains and Medium Strain Rates

**DOI:** 10.3390/polym10090988

**Published:** 2018-09-04

**Authors:** Francesca Carleo, Ettore Barbieri, Roly Whear, James J. C. Busfield

**Affiliations:** 1 School of Engineering and Materials Science, Queen Mary University of London, Mile End Rd, London E1 4NS, UK; f.carleo@qmul.ac.uk; 2 Japan Agency for Marine-Earth Science and Technology, Department of Mathematical Science and Advanced Technology (MAT), Yokohama Institute for Earth Sciences (YES) 3173-25, Showa-Machi, Kanazawa-Ku, Yokohama, Kanagawa 236-0001, Japan; e.barbieri@jamstec.go.jp; 3 Jaguar Land Rover, Banbury Road, Gaydon CV35 0RR, UK; rwhear1@jaguarlandrover.com

**Keywords:** viscoelastic behaviour, filled rubber, hysteresis, stress softening, review

## Abstract

Modelling the viscoelastic behavior of rubber for use in component design remains a challenge. Most of the literature does not consider the typical regimes encountered by anti-vibration devices that are deformed to medium dynamic strains (0.5 to 3.5) at medium strain rates (0.5/s to 10/s). Previous studies have either focused on the behaviour at small strains and small strain rates or in fast loading conditions that result in low cycle fatigue or tearing phenomena. There is a lack of understanding of the dynamic response of natural rubber suspension components when used in real vehicle applications. This paper presents a review of popular viscoelastic nonlinear constitutive models and their ability to model the mechanical behaviour of typical elastomer materials such as Natural Rubber (NR) incorporating different PHR (Parts per Hundred Rubber, XX) of carbon black. The range of strain and strain rate are typical for the materials used in rubber suspensions when operating in severe service operating conditions, such as over rough terrain or over pot-holes. The cyclic strain is applied at different amplitudes and different strain rates in this medium strain range. Despite the availability of many models in the literature, our study reports that none of the existing models can fit the data satisfactorily over a wide range of conditions.

## 1. Introduction

Research into the viscoelastic properties of rubberlike materials has attracted much attention particularly in the automotive [1,2,3,4] and biomedical sectors [5,6,7,8,9]. The observed properties include significant nonlinearity, hysteresis, cyclic stress softening and induced anisotropy during cyclic deformation.

Two different theoretical philosophies exist in formulating material models: the phenomenological and the micro-mechanical approach. The first is based on direct observation and the accurate measurement of the mechanical response of the rubber with curve-fitting of the experimental data or derivation of the strain energy function from a consideration of changes to the molecular conformation. The second, micro-mechanical approach, consists of the study of constituent materials and how they interact with each other [10,11]. The models can include atoms, molecules or the entire polymer chain. The multiscale nature of the polymers is reflected in this multiscale analysis, which can be based on different computational methods for specific length and time scales: quantum (∼$10^{-10}$ m, ∼$10^{-12}$ s), atomistic (∼$10^{-9}$ m, ∼$10^{-9}$–$10^{-6}$ s), mesoscopic (∼$10^{-6}$ m, ∼$10^{-6}$–$10^{-3}$ s) or macroscopic scale (∼$10^{-3}$ m, ∼1 s) [12].

The focus of this article is on a phenomenological model at the macroscale where the material is studied as homogeneous and continuous and the behaviour is governed by constitutive laws. Numerous, well-established hyperelastic models exist in the literature. A hyperelastic constitutive law generally consists of an equation relating the strain energy density to the three invariants of the strain tensor [13,14,15,16,17]. These models can predict the rubber behaviour in homogenous deformation states such tension, shear, compression or under a biaxial load with relatively few parameters. Even though hyperelastic models strictly apply only to time-independent deformation, they are often used in combination with viscoelastic models.

For dynamic applications, the reliability of using either a linear or a nonlinear viscoelastic theory depends on the level of deformation [18]. If the observed behaviour is independent of the degree of the applied stress or strains, it is possible to use a linear viscoelastic model where the shear modulus is described using a model such as a Prony series.

Linear fractional derivative models, such as Fractional Kelvin or Fractional Maxwell, are also popular because they describe the behaviour of viscoelastic dampers, in problems such as creep and relaxation, with a modest number of additional parameters [19,20,21,22].

However, filled rubber materials have a highly nonlinear viscoelastic behaviour [23]. Many of the nonlinear viscoelastic models have been developed under specific loading conditions such as: small strain, high strain rate, cyclic loading or when subjected to softening behaviour [24,25,26,27].

There are currently a number of nonlinear viscoelastic models proposed in the literature. This review will highlight the limitations of these existing models when predicting the real behaviour of a filled rubber.

**Figure 1:**
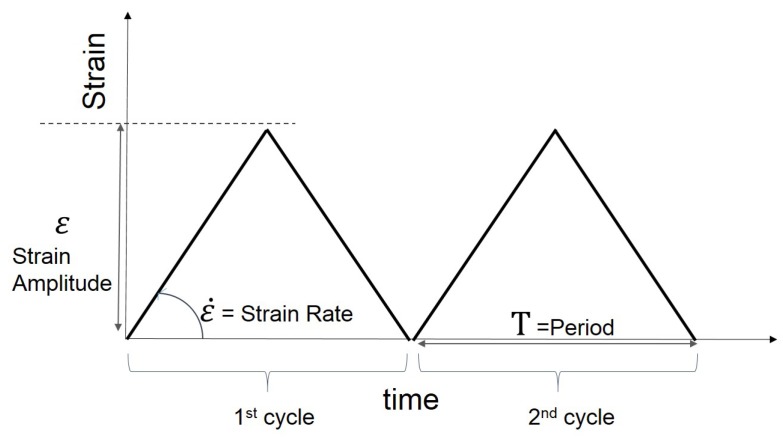
Schematic layout: Cyclic strain history with constant strain amplitude and strain rate (MAXAMP-CONST).

**Figure 2:**
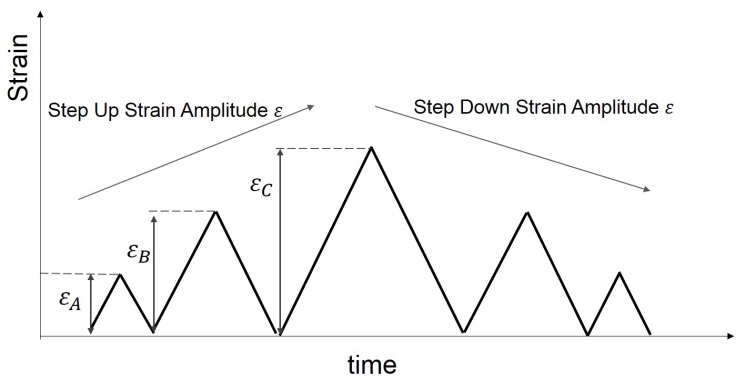
Schematic layout: Cyclic strain history with constant strain rate and various strain amplitudes organized in step up and step down (MAXAMP-UPDOWN).

**Figure 3:**
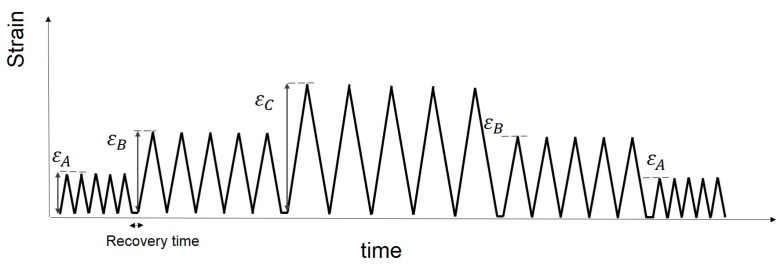
Schematic layout: Cyclic strain history with constant strain rate and various strain amplitudes organized in step up and step down. For each maximum strain amplitude the sample is loaded with five fully relaxing cycles. A recovery time is allowed after each series of five cycles. (MAXAMP-MIX).

In the present paper, Section 2 presents the experimental investigation. Cyclic uniaxial tensile tests were carried out to examine the nonlinear behaviour of several different vulcanised Natural Rubber (NR) compounds filled with different volume fractions of carbon black. The effect of the carbon black content on the materials behaviour is explored. In addition, the three separate strain history regimes have been adopted (Figure 1 MAXAMP-CONST, Figure 2 MAXAMP-UPDOWN and Figure 3 MAXAMP-MIX). Each allows a detailed analysis of different phenomena including hysteresis in the loading cycle, cyclic stress softening, recovery, permanent set and the effect of a pre-strain effect. Section 3 presents the basis of continuum mechanics and the methodology used to generate the parameters for specific material models. Section 4 shows a review of some of the phenomenological existing nonlinear viscoelastic models. The experimental data have been fitted to a range of models taken from the literature.

## 2. Experimental Methods

The literature reports various investigations into the experimental evidence for the nonlinear behaviour. The earlier works are on unfilled and filled Natural Rubber [28,29]. In the last few decades, the work has expanded into different materials, such as SBR, NBR, EPDM and polychloroprene [30,31,32,33,34].

### 2.1. Sample Preparation

In this paper, seven compounds supplied by TARRC (Tun Abdul Razak Research Centre, Brickendonbury, Hertford, UK), were examined. Each compound contained a different volume fraction of carbon black (FEF N550) that had been mixed into Natural Rubber (NR, SMR CV60). The compound formulations in phr (part per hundred of rubber by mass) are given in Table 1.

The curing conditions were supplied by TARRC and were confirmed using an oscillating die cure rheometer at a temperature of 150 ℃. Each material was vulcanised up to its t${}_{90}$ time.

### 2.2. Measurement Procedure

Uniaxial tension tests were obtained using an Electropulse Instron Test Machine. The cyclic loading, unloading and reloading tests were performed using four different strain rates of 0.5/s, 1.5/s, 3/s and 6/s. All the specimens have a gauge length of 12 mm and a cross section of 2 mm × 0.5 mm. The nominal strain, $\varepsilon^{NOM}$, was determined as the ratio of the axial displacement to the original length of a specimen (12 mm). The tensile force was measured by using a loading cell of 1 kN. The nominal stress, $\sigma^{NOM}$, was determined as the ratio of the axial force to the (undeformed) cross-sectional area of a specimen (2 mm × 0.5 mm) in the stress-free state.

Each of the materials were subjected to the loading conditions shown schematically in Figure 3 (MAXAMP-MIX). This test cyclically loaded each sample with five fully relaxing cycles up to 50% nominal strain, followed by five cycles to a strain of 100%, five cycles at 200% and finally five cycles up to a strain of 350%. Each specimen was then loaded in descending order, stepping down through each of the strain amplitudes. A recovery time of 5 s was allowed after each block of five cycles.

This test was difficult to undertake due to the large strain amplitude and the large strain rate being at the limit for the test machine. To reach the desired strain rates, the specimens needed to be short. In addition, the strain rate was too fast to be reliably calculated using the optical strain measuring device.

### 2.3. Experimental Observations

This section presents the experimental results of uniaxial extension tests under cyclic loading–unloading at a constant temperature carried on filled NR. The data highlights various features of the viscoelastic behaviour, including the strain-rate dependency, the Mullins effect and the pre-strain effect.

**Figure 4:**
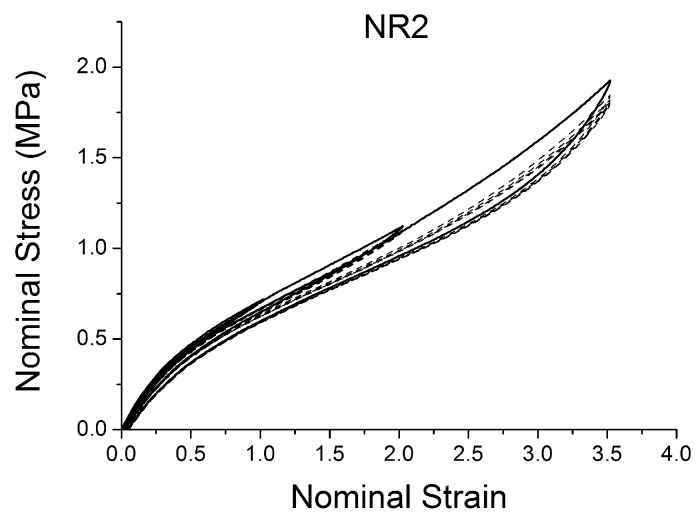
Stress–strain response of Natural Rubber filled with 2 phr of carbon black (NR2) submitted to cyclic uniaxial tension (Figure 3—MAXAMP-MIX) at 1.5/s. The solid curves are the trends for the first cycle at a given amplitude. The dotted curves are the trends after the initial cycle at given amplitude.

**Figure 5:**
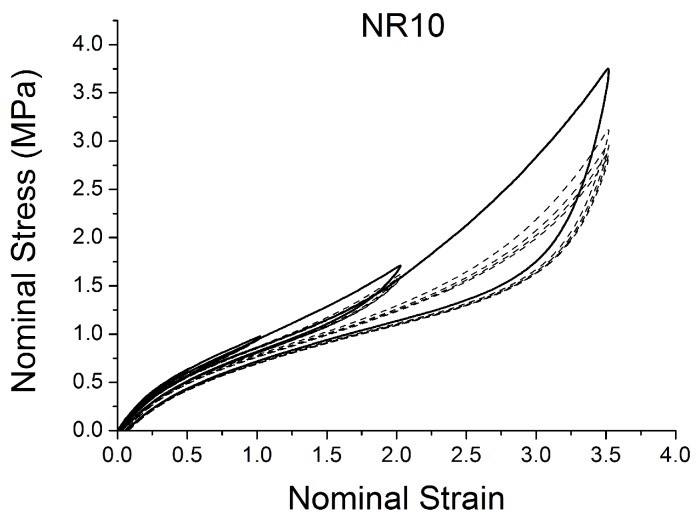
Stress–strain response of Natural Rubber filled with 10 phr of carbon black (NR10) submitted to cyclic uniaxial tension (Figure 3—MAXAMP-MIX) at 1.5/s. The solid curves are the trends for the first cycle at a given amplitude. The dotted curves are the trends after the initial cycle at given amplitude.

**Figure 6:**
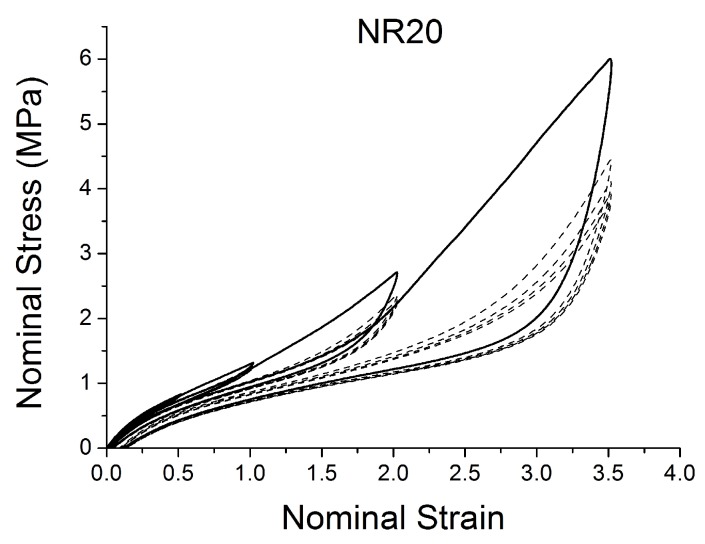
Stress–strain response of Natural Rubber filled with 20 phr of carbon black (NR20) submitted to cyclic uniaxial tension (Figure 3—MAXAMP-MIX) at 1.5/s. The solid curves are the trends for the first cycle at a given amplitude. The dotted curves are the trends after the initial cycle at given amplitude.

**Figure 7:**
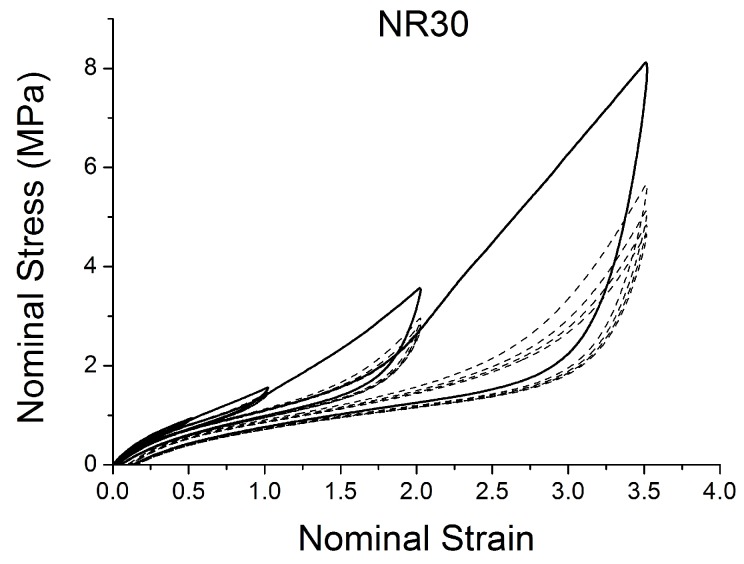
Stress–strain response of Natural Rubber filled with 30 phr of carbon black (NR30) submitted to cyclic uniaxial tension (Figure 3—MAXAMP-MIX) at 1.5/s. The solid curves are the trends for the first cycle at a given amplitude. The dotted curves are the trends after the initial cycle at given amplitude.

**Figure 8:**
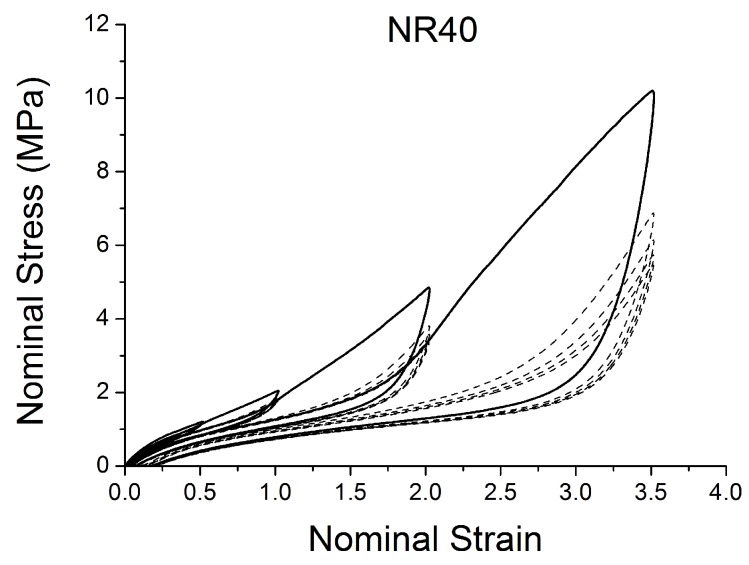
Stress–strain response of Natural Rubber filled with 40 phr of carbon black (NR40) submitted to cyclic uniaxial tension (Figure 3—MAXAMP-MIX) at 1.5/s. The solid curves are the trends for the first cycle at a given amplitude. The dotted curves are the trends after the initial cycle at given amplitude.

**Figure 9:**
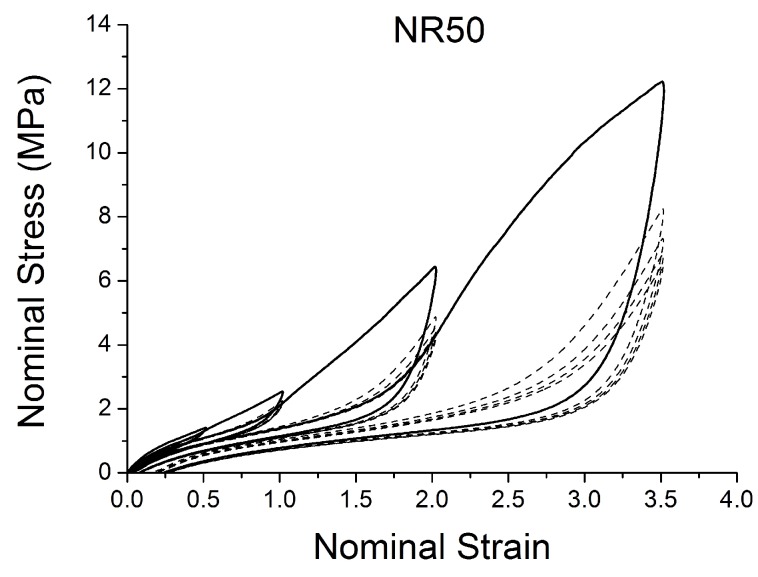
Stress–strain response of Natural Rubber filled with 50 phr of carbon black (NR50) submitted to cyclic uniaxial tension (Figure 3—MAXAMP-MIX) at 1.5/s. The solid curves are the trends for the first cycle at a given amplitude. The dotted curves are the trends after the initial cycle at given amplitude.

**Figure 10:**
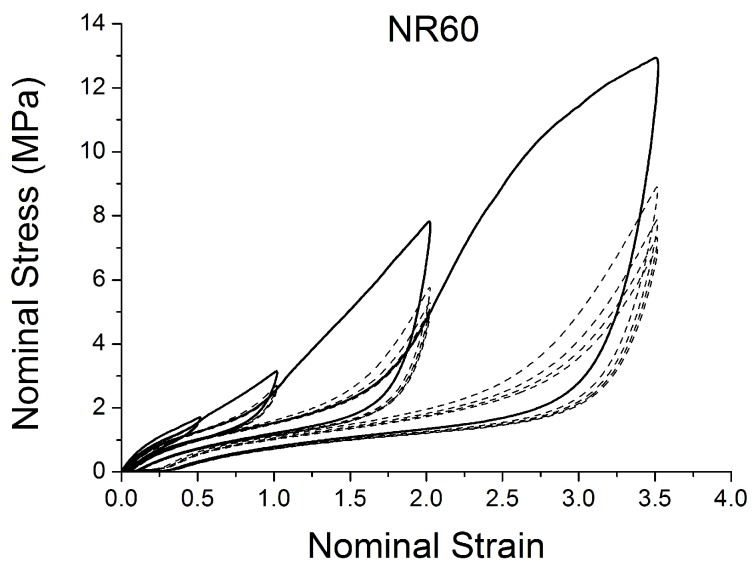
Stress–strain response of Natural Rubber filled with 60 phr of carbon black (NR60) submitted to cyclic uniaxial tension (Figure 3—MAXAMP-MIX) at 1.5/s. The solid curves are the trends for the first cycle at a given amplitude. The dotted curves are the trends after the initial cycle at given amplitude.

**Figure 11:**
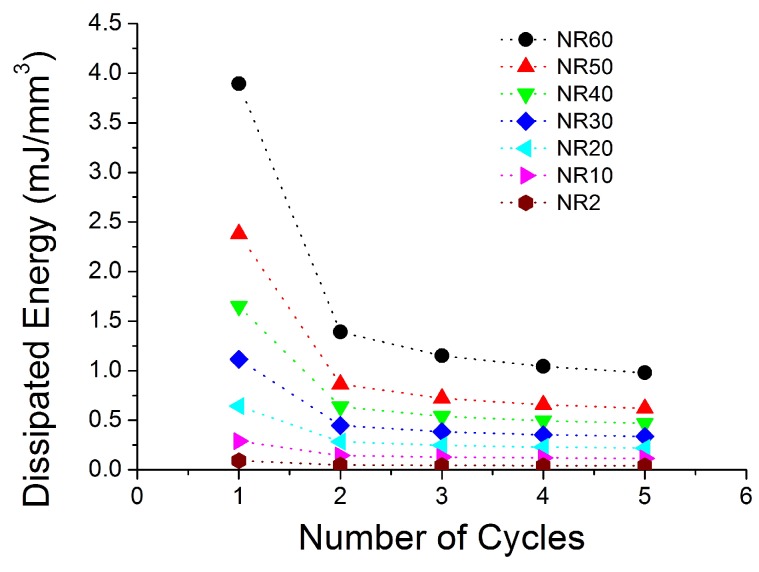
Dissipated energy at different cycles for all seven compounds submitted to cyclic uniaxial tension (Figure 1—MAXAMP-CONST) with maximum nominal strain 2 at 3/s.

**Figure 12:**
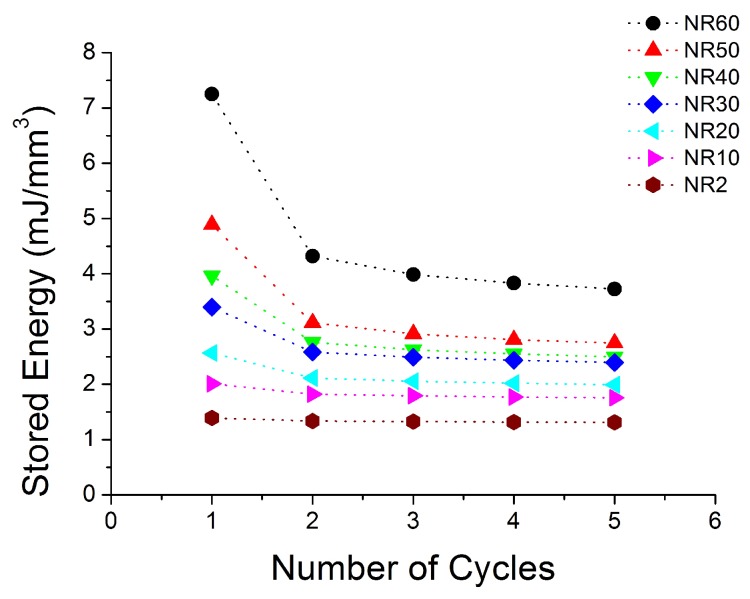
Loading energy at different cycles for all seven compounds submitted to cyclic uniaxial tension (Figure 1—MAXAMP-CONST) with a maximum nominal strain 2 at 3/s.

**Figure 13:**
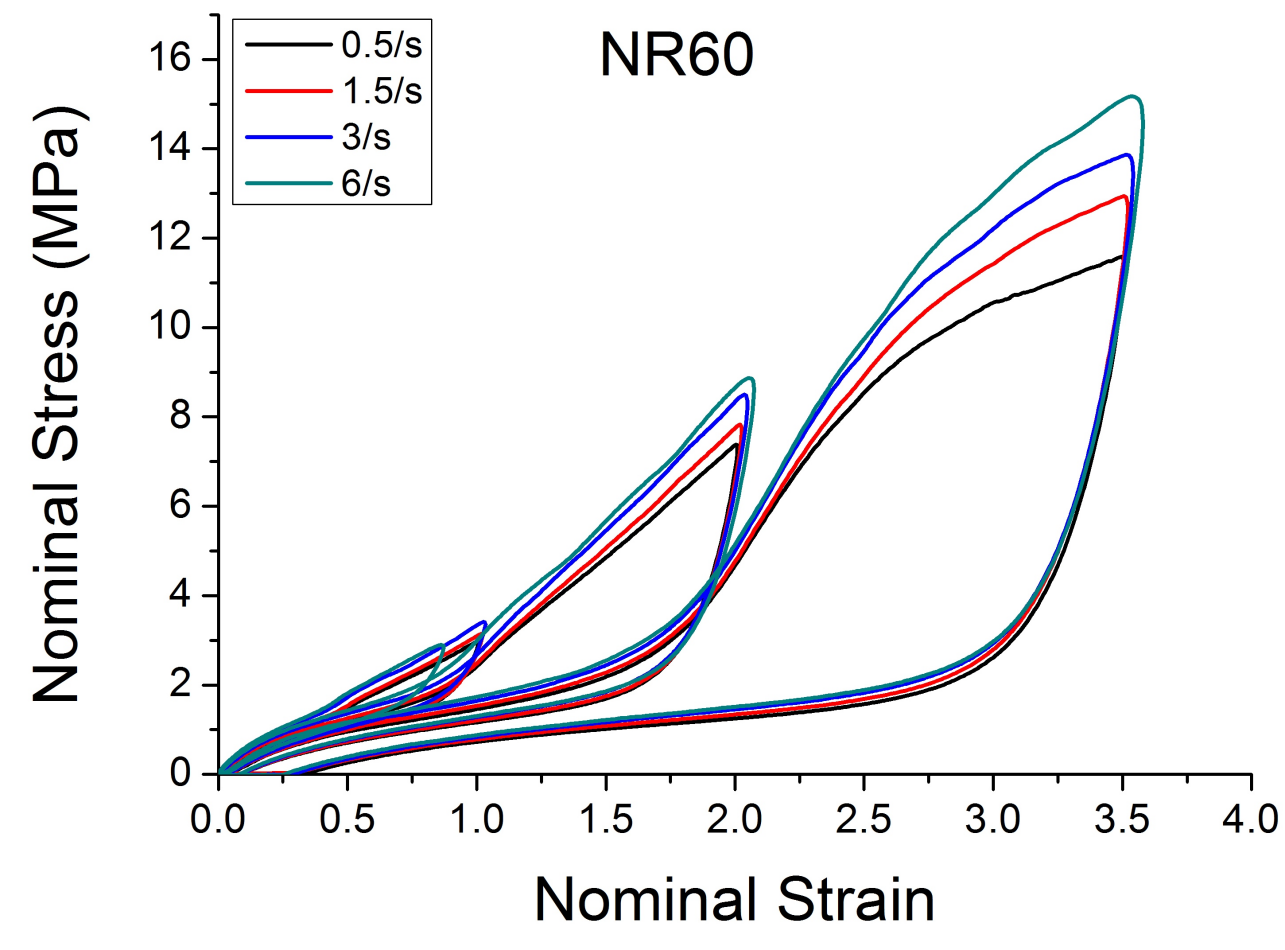
Stress–strain response of Natural Rubber filled with 60 phr of carbon black (NR60) submitted to cyclic uniaxial tension (Figure 3—MAXAMP-MIX) at four different strain rates with maximum strains ε = 1, ε = 2 and ε = 3.5.

**Figure 14:**
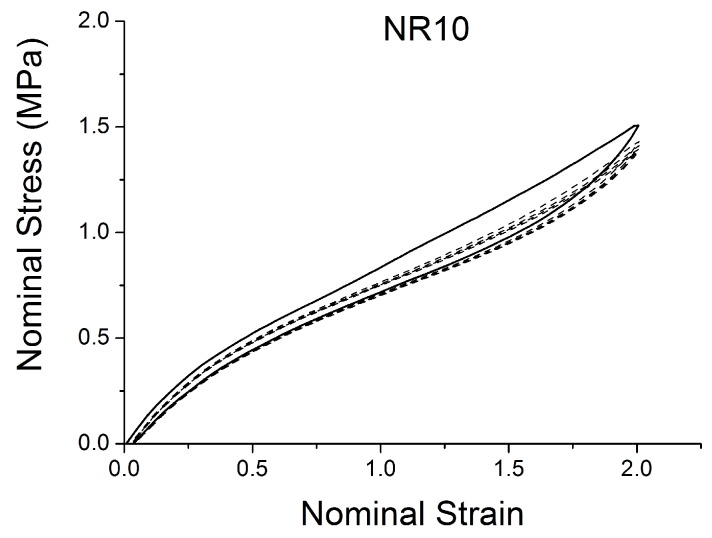
Stress–strain response of Natural Rubber filled with 10 phr of carbon black (NR10) submitted to cyclic uniaxial tension (Figure 1—MAXAMP-CONST) with maximum nominal strain 2. The solid curves are the trends for the first cycle at a given amplitude. The dotted curves are the trends after the initial cycle at a given amplitude.

**Figure 15:**
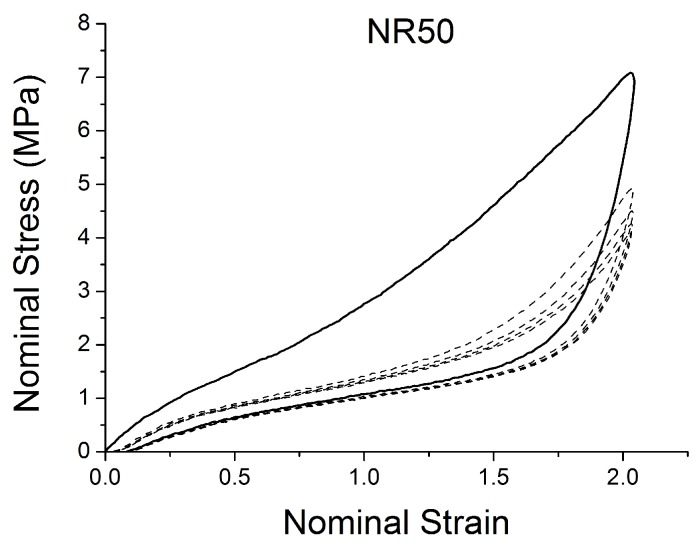
Stress–strain response of Natural Rubber filled with 50 phr of carbon black (NR50) submitted to cyclic uniaxial tension (Figure 1—MAXAMP-CONST) with maximum nominal strain 2. The solid curves are the trends for the first cycle at a given amplitude. The dotted curves are the trends after the initial cycle at a given amplitude.

**Figure 16:**
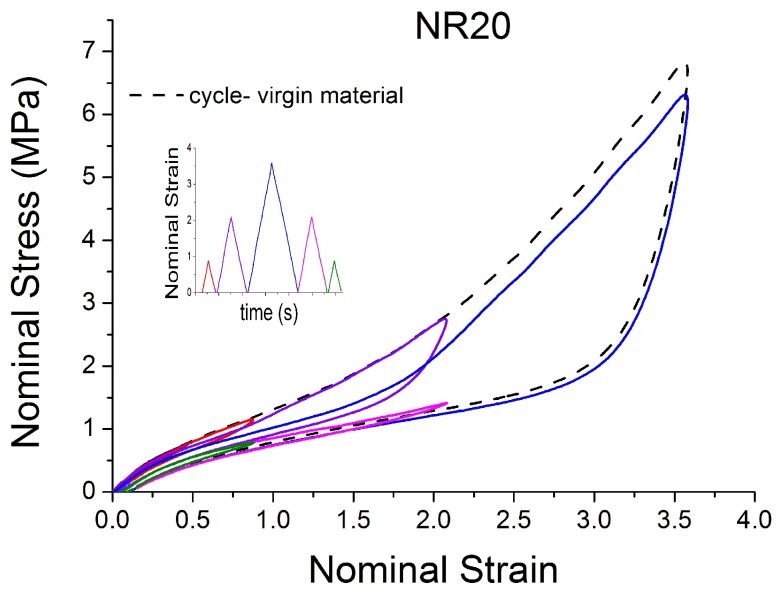
Stress–strain response of Natural Rubber filled with 20 phr of carbon black (NR20) submitted to cyclic uniaxial tension (Figure 2—MAXAMP-UPDOWN). The different behavior of the material after a different pre-strain is evident.

Figure 4, Figure 5, Figure 6, Figure 7, Figure 8, Figure 9 and Figure 10 show the results as nominal stress versus nominal strain. The solid lines are the trends for the first cycle at a given amplitude. The dotted lines are the trends after the initial cycle at given amplitude. They allow the following comments:

- Natural Rubber filled with just 2 phr of carbon black (NR2) shows a behaviour that is the most elastic (Figure 4). The hysteresis, estimated as the area between the loading and the unloading stress–strain paths, increases with the amount of carbon black in the compound (Figure 11).
- There is a reduction in the stress on each successive loading at the same strain amplitude. The reduction is largest between the first and second loading-unloading cycles and becomes less significant in the following cycles (Figure 12). The effects of stress softening are less significant for compounds with lower CB content.
- There are residual strains, known as the permanent set, that increase with the amount of carbon black and that depend on the maximum applied strain.
- The behaviour of rubber is rate dependent, with an enhancement of stress when the deformation rate is increased (Figure 13).
- The unloading and reloading strain-stress path responses differ (Figure 14 and Figure 15).
- The material approaches to the virgin loading path whenever the load is increased beyond its previous maximum value (Figure 16).
- The stress–strain path is highly dependent on the loading history (Figure 16). In the following sections, this phenomenon is referred to as the pre-strain effect.

Because of the large strains encountered during this testing (especially with a large amount of carbon black, Figure 10 and Figure 13), the specimens tend to slip from the clamps during dynamic tensile testing. This may explain why the curves at high filler and high strain have an ‘artificial’ extra inflexion point (where the behaviour appears to soften unexpectedly). The testing difficulty may be part of the reason why there appears to be little data reported in the literature on the tensile behaviour under medium strain and medium strain rate.

## 3. Preliminary Remark

### 3.1. Basics of Continuum Mechanics

Continuum mechanics is a powerful tool to explain various physical phenomena without detailed knowledge of their complex micro-structure. A basic assumption states that a body *B* may be modelled as a continuum distribution of matter in space and time and it is imagined as a composition of material points or particles [35,36]. If we consider a material body *B* of the three-dimensional Euclidean space, each material particle P∈B corresponds with a geometrical point. The position vector X labels the particle *P* at a given time *t* in the reference (or undeformed) configuration. When the region moves to a new configuration, known as current or deformed, the particle *P* corresponds to the new position x. The state of deformation at each material point is described by a deformation gradient F, Equation (1). Other important tensors that are useful to describe the deformation of the material are the left Cauchy–Green deformation tensor B, Equation (2) and the right Cauchy–Green deformation tensor C, Equation (3), both obtained by multiplying F by its transpose:

(1) $$F=\frac{\partial x}{\partial X},$$

(2) $$B=FF^{T},$$

(3) $$C=F^{T}F.$$

Equations (4)–(6) define the three invariants of B:

(4) $$I_{1}=tr\left(B\right)=\lambda_{1}^{2}+\lambda_{2}^{2}+\lambda_{3}^{2},$$

(5) $$I_{2}=\frac{\left[I_{1}^{2}-tr\left(B\right)\right]}{2}=\lambda_{1}^{2}\lambda_{2}^{2}+\lambda_{2}^{2}\lambda_{3}^{2}+\lambda_{3}^{2}\lambda_{1}^{2},$$

(6) $$I_{3}=\det\left(B\right)=\lambda_{1}\lambda_{2}\lambda_{3},$$

where $\lambda_{i}$ are the principal stretch ratios oriented along the directions of three axes in the coordinate systems. It is reasonable to assume that rubber-like materials are incompressible, which results in $I_{3}$ = 1.

For the uniaxial test of an incompressible material, the principal stretches are given by Equation (7) and the deformation gradient is given by Equation (8):

(7) $$\lambda_{1}=\lambda \;\;\;\lambda_{2}=\lambda_{3}=\frac{1}{\sqrt{\lambda }},$$

(8) $$F=\left[\begin{array}{ccc} \lambda & 0 & 0\\ 0 & \frac{1}{\sqrt{\lambda }} & 0\\ 0 & 0 & \frac{1}{\sqrt{\lambda }} \end{array}\right].$$

### 3.2. Determination of Model Parameters and Correlation Matrix

Assuming that there is a constitutive model $f(x_{i},k_{1},\cdots ,k_{n})$ with $x_{i}$ the input data set, usually the strain, and $k_{j}$ (with j=1,…,n) the material parameters, the model $f(x_{i},k_{1},\cdots ,k_{n})$ was fitted with an optimization algorithm that minimized the sum *S* of the squares of the residuals $r_{i}$ of the experimental data points $y_{i}$ from the model.:

(9) $$r_{i}=y_{i}-f\left(x_{i},k_{1},\cdots ,k_{n}\right),$$

(10) $$S=\sum_{i=0}^{m}r_{i}^{2}.$$

In addition, a Sensitivity Analysis was conducted on some models. A variance-based analysis is one of the most accurate techniques for these nonlinear theories [37]. It allows the full exploration of the input space (global method) and the estimation of the covariance matrix provides an exploratory study of the inter-relationship between variables (how the variables change in relation to one another).

## 4. Nonlinear Viscoelastic Models

Different models exist for different loading conditions to account for the strong nonlinearity of the filled vulcanized rubber.

For the case of a cyclic loading condition with small strain amplitude, the filled rubber shows a dependence of the viscoelastic storage modulus on the magnitude of the applied strain. This effect is known as the Fletcher–Gent effect or the Payne effect and this has been extensively studied previously [4,26,38,39]. One of the modelling techniques is based on the use of the fractional derivatives [40,41]. In particular Lion, [40], proposed a model with six parameters, which combines the fractional Maxwell model with the concept of an intrinsic time z(t). Such time is a phenomenological measure for the current state of the material’s microstructure.

A finite strain visco-plastic-elastic model for rubber with reinforcing filler was proposed by Osterlof [26,27]. It assumes small strains and can capture the amplitude dependency and frequency dependency with a multiplicative split of the deformation gradient into elastic, plastic and viscoelastic components.

Another important phenomenon is the Mullins effect, which is observed at larger strains. It includes the cyclic stress softening (Figure 15) and the ability of the material to recover and return towards the virgin stress–strain path when the load rises over the previous maximum applied stress value (Figure 16). Many experimental data show that the Mullins effect is strongly anisotropic and produces a significant permanent set [31,42,43].

The next section describes in detail some models that can be used to characterise some of these nonlinear effects (Figure 4, Figure 5, Figure 6, Figure 7, Figure 8, Figure 9 and Figure 10). In particular, the focus is on the ability to predict the Mullins Effect, the different unloading and reloading curves and the pre-strain effect.

### 4.1. Phenomenological Models

The phenomenological models can roughly be classified into three categories: the damage models, the rheological models with serial and parallel combination of elastic and viscous elements and the constitutive equations based on a rubber elasticity model.

#### 4.1.1. Damage Models

Viscoelastic damage models use a softening parameter, called the damage variable. The hyperelastic strain energy density $W_{0}$ is penalised by a damage function *D*, depending on the maximum deformation, Equation (11):

(11) $$W=\left(1-D\right)W_{0}\;\mathrm{with}\;\;D\in \left[0,1\right].$$

Simo [44] was the first to adopt such a continuum damage mechanics theory. Over the subsequent years, various models have been defined [32,45,46]. In these works, the damage is not related to physical considerations, but it is purely phenomenological. Ogden and Roxburgh [45] proposed a modified version of the strain energy W(F) [35], which they called pseudo-elastic model W(F, η), to describe the Mullins Effect, Equation (12):

(12) $$W\left(F,\eta \right)=\eta W_{0}(F)+\phi \left(\eta \right).$$

The damage parameter η (Equation (13)) is activated when the unloading is initiated and inactivated when, during the reloading, the strain becomes larger than the previous maximum value. The variable η reaches the minimum value $\eta_{m}$ when the material is fully unloaded. ϕ(η) is reported as the damage function and $\phi (\eta_{m})$ may be interpreted as the measure of the energy required to cause the damage in the material. Such model, however, neglects residual strains.

The isotropic pseudoelastic model proposed by Dorfmann and Ogden [32] introduced a residual strain by introducing a second damage function $W(F,\eta_{1},\eta_{2})$ (Equation (14)).

More recently, Wrubleski [46,47,48] has proposed modified versions of the Dorfmann damage $\eta_{1}$ to increase fitting capability (Equation (15)):

(13) $$\eta_{Ogden}=1-\frac{1}{r}\mathrm{erf}\left(\frac{W_{dev}^{max}-W_{dev}}{m}\right),$$

(14) $$\eta_{1Dorfmann}=1-\frac{1}{r}\tanh\left(\frac{W_{dev}^{max}-W_{dev}}{m}\right),$$

(15) $$\eta_{Wrubleski}=1-\frac{1}{r}{\left(\tanh\left(\frac{W_{dev}^{max}-W_{dev}}{m}\right)\right)}^{q}.$$

$W_{dev}^{max}$ is the maximum deviatoric strain energy, $W_{dev}$ is the deviatoric strain energy and r,mandq are the material parameters.

In their work, Guo and Sluys [49] discussed limitations and advantages of the continuum mechanics model and the pseudo-elastic model.

**Figure 17:**
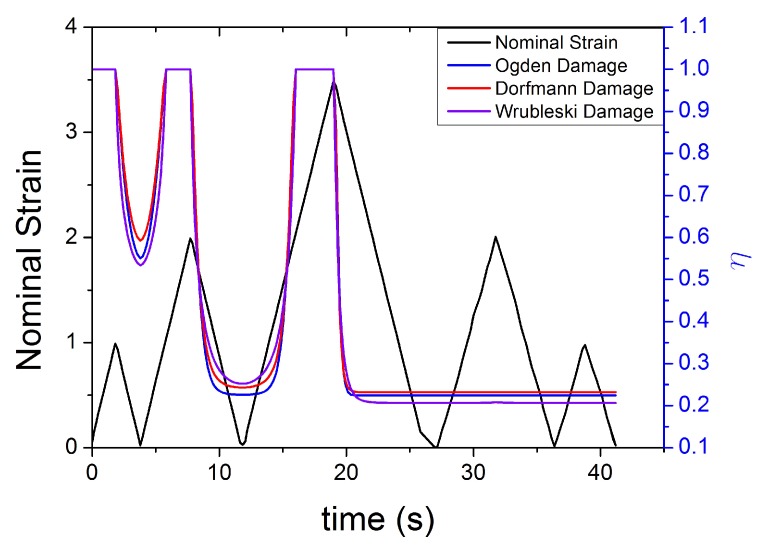
Theoretical evolution of the damage function for three different models [32,45,46] for NR60 with cyclic uniaxial stress with different maximum strain amplitude in step up and step down phases (MAXAMP-UPDOWN Figure 3).

**Figure 18:**
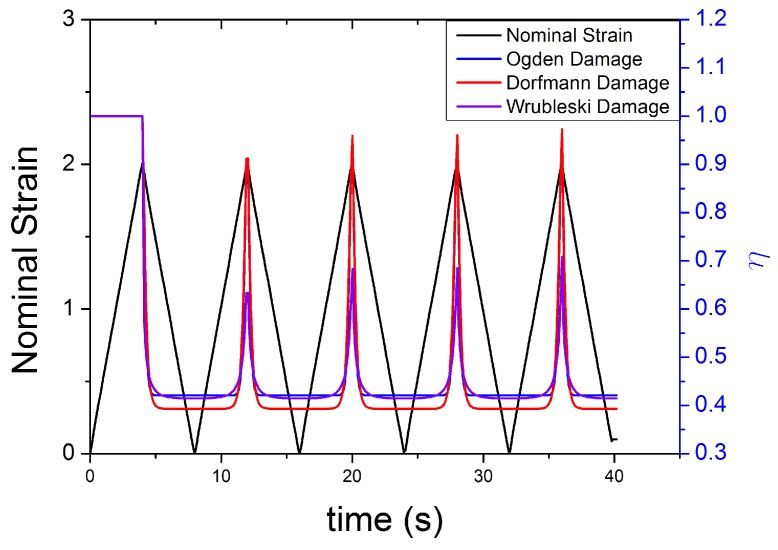
Theoretical evolution of the damage function for three different models ([32,45,46] for NR60 with cyclic uniaxial test with uniform maximum strain amplitude (MAXAMP-CONST Figure 1).

The evolution of damage parameters for Ogden [45], Dorfmann [32] and Wrubleski [48] is plotted in Figure 17 and Figure 18, for two different input strain histories: cyclic tensile stretch at different strain amplitudes (MAXAMP-UPDOWN) and cyclic tensile stretch at constant amplitude cyclic strain (MAXAMP-CONST). Experimental data of Natural Rubber with 60 phr of carbon black are used to fit the models and compare the results following the time history of the damage parameter. This approach is useful as it helps examine the limitations of such models in predicting phenomena such as the Mullins effect and the cyclic stress relaxation.

The variable η, in the analysed conditions, does not show qualitative differences from one definition to another.

**Figure 19:**
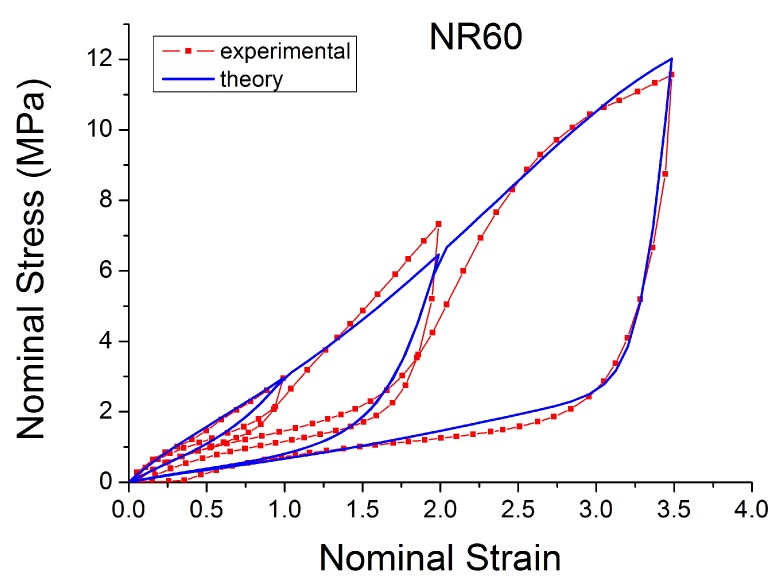
Comparison of Ogden model [45] with experimental data when the specimen of NR60 is loaded with cyclic uniaxial stress with different maximum strain amplitude in step up and step down phases (MAXAMP-UPDOWN Figure 3).

**Figure 20:**
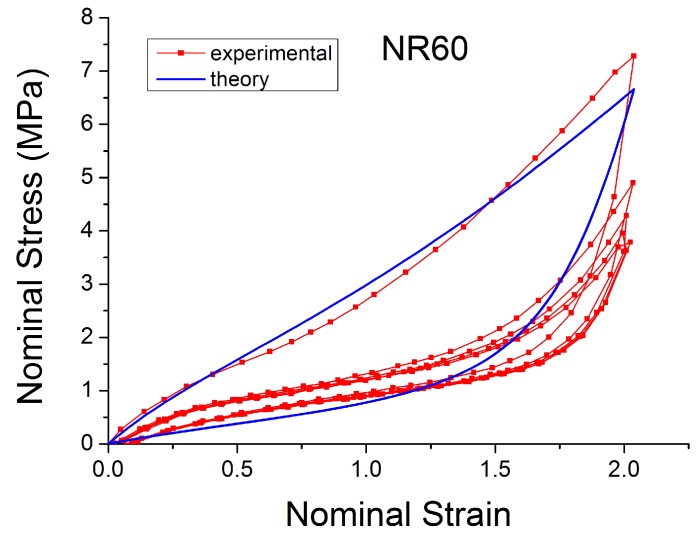
Comparison of Ogden model [45] with experimental data when the specimen of NR60 is loaded with cyclic uniaxial stress with uniform maximum strain amplitude (MAXAMP-CONST Figure 1).

The theoretical stress–strain response returns to the virgin stress–strain path without any transition (Figure 19). The unloading and reloading (for a new cycle) paths are on the same curve, which disagrees with the experiments when, for example, after a large strain the material is reloaded at a smaller amplitude. In addition, these laws can not reproduce the hysteresis during these cycles. Furthermore, the models fail to capture the stress–strain curve for cyclic uniaxial stress with uniform maximum strain amplitude, Figure 20. The theoretical stress for the maximum strain is the same for the first and following loadings, which ignores the cyclic stress-softening. The hysteresis for the cycles after the first is also ignored.

**Figure 21:**
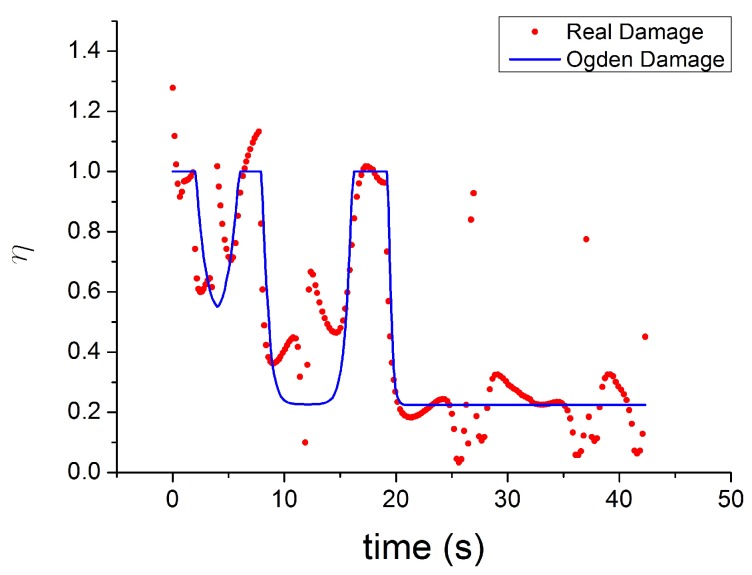
Comparison of the evolution of theoretical softening function [45] with a real damage parameter estimate as the ratio between the experimental stress and the hyperelastc stress for NR60 with cyclic uniaxial stress with different maximum strain amplitude in step up and step down phases MAXAMP-UPDOWN Figure 3).

**Figure 22:**
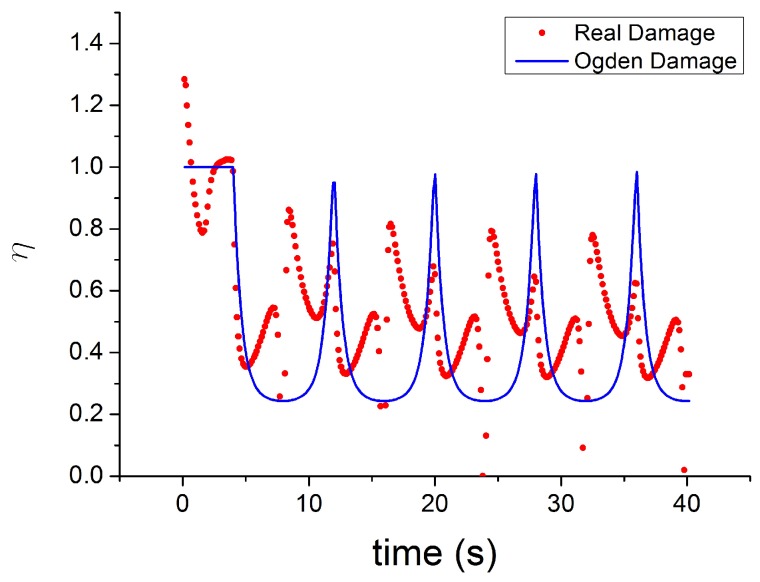
Comparison of the evolution of theoretical softening function [45] with real damage parameter estimate as a ratio of the experimental stress to the hyperelastic stress for NR60 with cyclic uniaxial test with uniform maximum strain amplitude (MAXAMP-CONST Figure 1.

The limitations of these models are clear when the theoretical softening function [45] is compared with the real damage estimated as ratio between the experimental stress and the Yeoh model fitted for the first loading curve (Figure 21 and Figure 22). The real damage can be considered also to be the ratio of the experimental stress in a subsequent cycle to the experimental stress at the same strain during the first loading curve. It is possible to see that the real experimentally observed damage function shows the following features:

- It has no symmetry during the unloading and reloading phase of each cycle.
- It is not constant after significant peak strain when the material is reloaded to a smaller strain.
- It never returns to the value 1 (there is always a softening when the material is reloaded even at a fixed maximum strain amplitude).

In Figure 21 and Figure 22, a conflict between the theory and experimental data is evident, which has already been highlighted by Diani [50]. The damage function must be increasing monotonically during the loading to satisfy the Clausius–Duhem inequality. However, the experimental results indicate that the softening variable shows a clear non-monotonic trend.

#### 4.1.2. Additive Split of the Stress

Some of the viscoelastic models split the stress into an equilibrium part that is rate-independent and an additional viscous part. This approach assumes a multiplicative decomposition of the deformation gradient into an elastic and inelastic time dependent part [27,38,51,52,53,54]:

(16) $$\sigma =\sigma_{equil}+\sigma_{viscous}.$$

**Figure 23:**
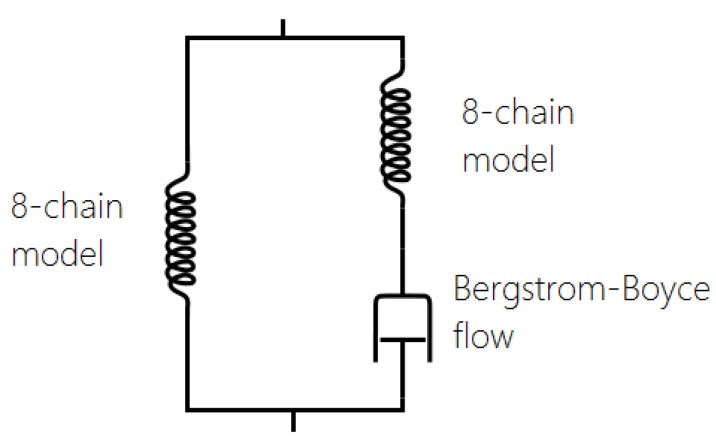
Rheological model: hyperelastic model in parallel with viscoelastic network with Bergstrom Boyce flow [51].

**Figure 24:**
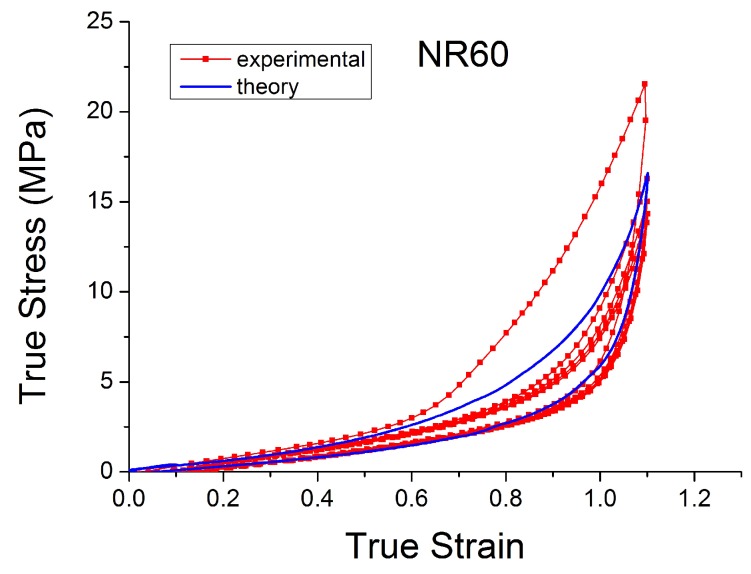
Comparison of Bergstrom Boyce model [51] with experimental data for NR50 in cyclic uniaxial test.

One of the most widely adopted models is the micro-mechanism inspired model proposed by Bergstrom and Boyce [51,55,56,57], which is already widely implemented in various Finite Element Software, such as ABAQUS. They introduced a strain rate law (${\dot{\varepsilon }}_{cr}$ ) (Equation (17)) on the assumption that the mechanism responsible for the time-dependent behaviour is the reptation of macromolecules that are elastically inactive. It is a hyper-viscoelastic model (with a superposition of viscoelastic and hyperelastic networks in parallel, Figure 23) that is able to predict hysteresis and strain rate dependence for slowly applied loads. It has a remarkable correspondence to the equilibrium state after many cycles (Figure 24).

(17) $${\dot{\varepsilon }}_{cr}=A{\left(\lambda_{cr}-1+E\right)}^{C}\sigma^{m}.$$

A,E,Candm are material parameters, λ is the stretch ratio and σ the Cauchy stress.

**Figure 25:**
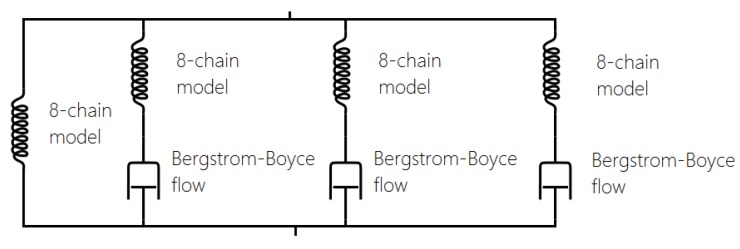
Parallel Rheological Framework (PRF): hyperelastic model in parallel with three viscoelastic networks with Bergstrom Boyce flow [51].

**Figure 26:**
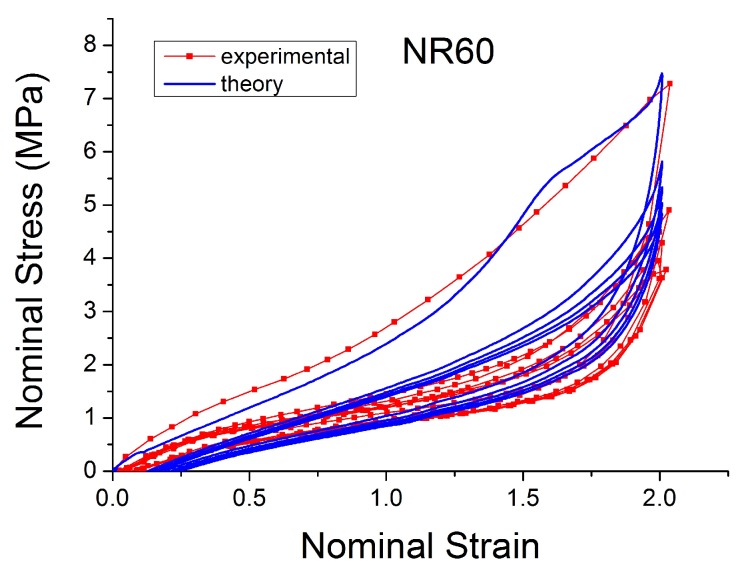
Comparison PRF model with experimental data for NR60 in cyclic uniaxial test.

In the last decade, a finite-strain constitutive framework known as a Parallel Rheological Framework ([23,58]) has been developed to model nonlinear viscoelasticity, Mullins Effect and a permanent set in elastomers. It consists of multiple networks connected in parallel (Figure 25). An arbitrary number of viscoelastic networks are used in parallel with an equilibrium network that can be purely elastic or elastoplastic. Figure 26 shows the efficiency of this framework (with three viscoelastic networks in parallel with a hyperelastic network, Figure 25). The model fits well with the experimental response of Natural Rubber filled with 60 phr carbon black under cyclic loading at uniform strain amplitude. However, the model introduces a non-realistic shape in the first loading path. In addition, this approach requires a significant number of parameters that are difficult to derive and which change erratically with small changes in the input test data.

**Figure 27:**
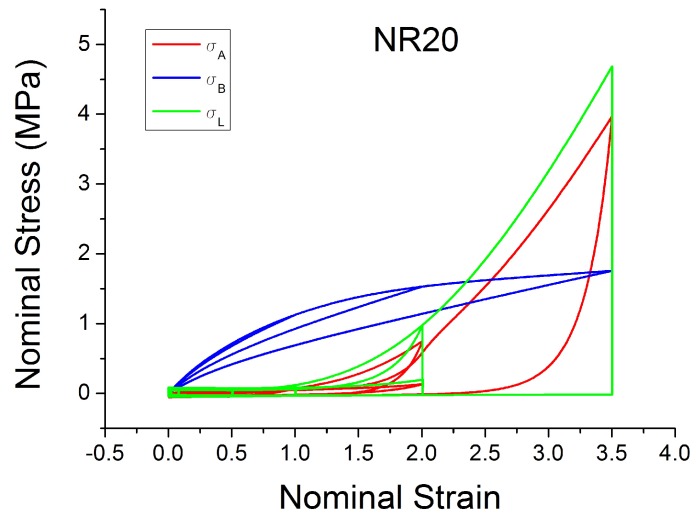
Additive, Basic and Limiting stress defined in the MORPH model [59] and fitted for NR20 loaded with cyclic uniaxial stress with different maximum strain amplitude in step up and step down phases (MAXAMP-UPDOWN, Figure 2).

**Figure 28:**
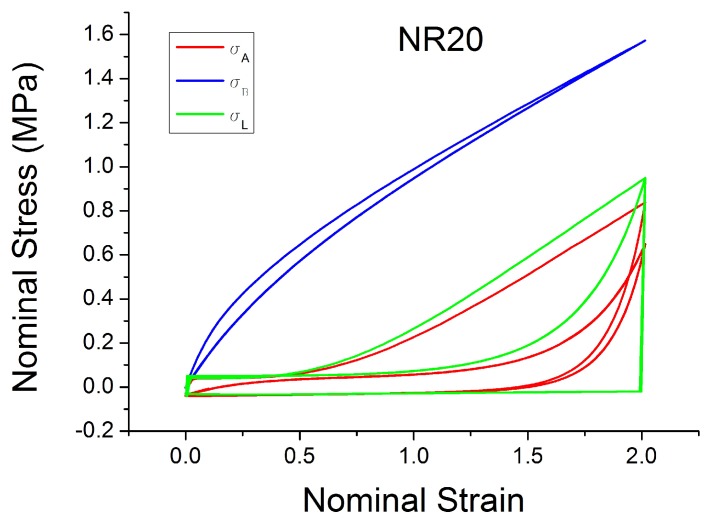
Additive, Basic and Limiting stress defined in the MORPH model [59] and fitted for NR20 loaded with cyclic uniaxial test with uniform maximum strain amplitude (MAXAMP-CONST, Figure 1).

Besdo and Ihlemann [59] proposed an innovative phenomenological inelastic constitutive model (MORPH model) based on an additive split of the stress (Equation (18)) of a basic stress $\sigma_{B}$ (Equation (19)) and an additional stress $\sigma_{A}$, which is defined by the stress rate (Equation (20)). It is based on the idea of approximating the loading cycles by a limiting line $\sigma_{L}$ (Equation (21)), which acts as asymptotic curve for the stress–strain response (Figure 27 and Figure 28). The parameters α, β and γ are defined in Equation (22). The MORPH model is defined by the characterization of 8 ($p_{i}$) material parameters (assuming incompressible behaviour) and by introducing a history function $B_{T}^{H}$. B is the left Cauchy strain and $B_{T}$ is a special invariant given by the maximum difference of two eigenvalues.

**Figure 29:**
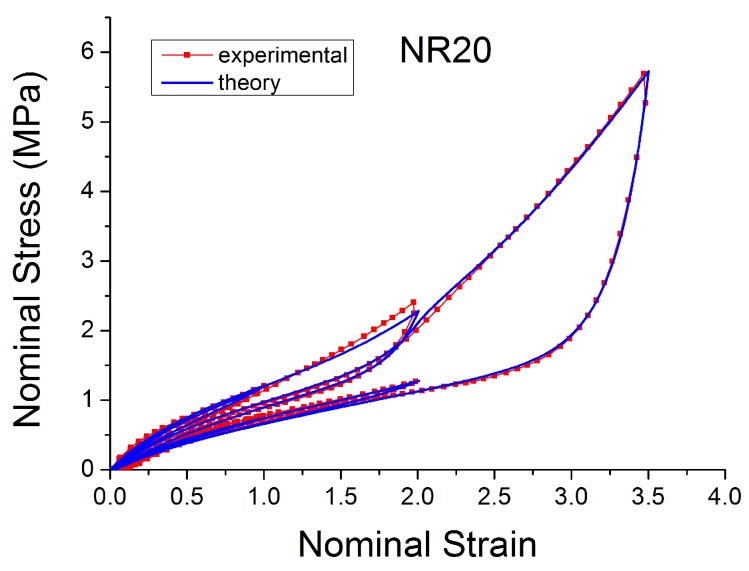
Comparison MORPH model [59] with experimental data for NR20 loaded with cyclic uniaxial stress with different maximum strain amplitude in step up and step down phases (MAXAMP-UPDOWN, Figure 2).

**Figure 30:**
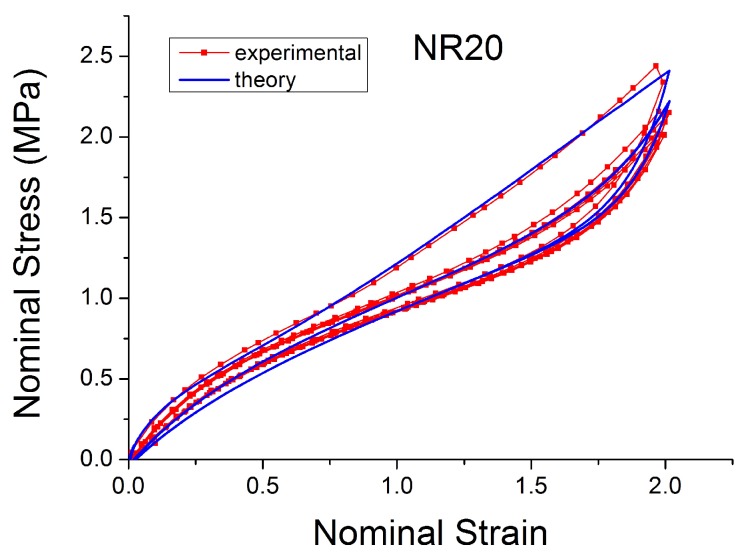
Comparison MORPH model [59] with experimental data for NR20 loaded with cyclic uniaxial test with uniform maximum strain amplitude (MAXAMP-CONST, Figure 1).

**Figure 31:**
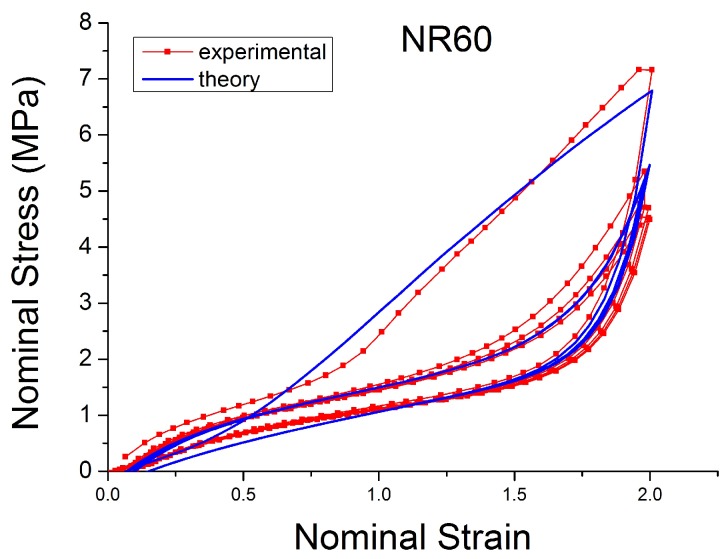
Comparison MORPH model [59] with experimental data for NR60 loaded with cyclic uniaxial test with uniform maximum strain amplitude (MAXAMP-CONST, Figure 1).

The model fits the hysteresis and the influence of the loading history for rubber very well for materials with a small amount of filler, as is shown in Figure 29. A good fit is also shown for the cyclic uniaxial cyclic test at constant strain amplitude. The model can predict the stress softening between the first and the second cycle (Figure 30). However, the model is less able to work well when the amount of filler is increased. Figure 31 shows an unrealistic effect, especially in the area of small strain. In addition, a detailed sensitivity analysis shows strong correlations existing between the various parameters $p_{i}$, with i=1,…,8.

In 2010, Freund [60] proposed a new algorithm for the 3D generalization of one-dimensional model and tested the algorithm on the MORPH model. The idea is based on the concept of representative directions. It is a continuum mechanics generalization without any reference to the physical structure and applies to any model, even to inelastic materials without a known free energy:

(18) $$\sigma =\sigma_{B}+\sigma_{A},$$

(19) $$\sigma_{B}=2\alpha B,$$

(20) $$\dot{\sigma_{A}}=\beta \dot{B_{T}}\left(\sigma_{L}-\sigma_{A}\right),$$

(21) $$\sigma_{L}=\gamma \exp\left(p_{7}\frac{\dot{B}}{\dot{B_{T}}}\frac{B_{T}}{B_{T}^{H}}\right)+p_{8}\frac{\dot{B}}{\dot{B_{T}}},$$

(22) $$\alpha =p_{1}+\frac{p_{2}}{\sqrt{1+{\left(p_{3}B_{T}^{H}\right)}^{2}}};\beta =\frac{p_{4}}{\sqrt{1+{\left(p_{3}B_{T}^{H}\right)}^{2}}};\gamma =p_{5}B_{T}^{H}\left(1-\frac{p_{6}}{\sqrt{1+{\left(p_{6}^{2}B_{T}^{H}\right)}^{2}}}\right).$$

A different approach was proposed by Liu [54]. This model is based on uniaxial test data and assumes that the materials is isotropic and incompressible and deforms in an isothermal process. The total stress is the sum of a primary hyperelastic stress and an additional viscoelastic stress term. The main response is taken as the average stress–strain response in the hysteresis produced during cycling loading, which changes with strain amplitude and rate. The additional viscoelastic stress is the difference between the loading stress and the primary stress. The model by Liu can predict the steady state response after multiple loading cycles.

In 2012, a different approach was proposed by Rickaby [61]. He developed a model able to reproduce the inelastic behaviours (cyclic stress softening, Mullins effect, residual strain) for a uniaxial tension of an isotropic rubber that utilised a large number of parameters. In this model inelastic features can be either excluded or added without compromising the integrity of the model.

#### 4.1.3. Constitutive Laws Based on a Rubber Elasticity Model

Several researchers have sought a physical understanding of the nonlinear and inelastic behaviour of reinforced elastomers. An alternative to the damage or rheological models is given by the model derived from micromechanical observations. The rubber-like materials are treated as a network of long chains connected by links [62]. The types of behaviour are considered to be a consequence of the molecular motion of these polymer chains and the breakage and reformation of the links.

Kluppel [63,64] introduced a microstructure based model of filled rubber known as Dynamic Flocculation Model (DFM). During vulcanisation, the filler particles flocculate together to form clusters. The constitutive hypothesis of DFM is derived from the thermodynamic equilibrium state (at an infinitely slow rate of deformation). The stress response of filled rubber can be derived by two mesoscopic phenomena: hydrodynamic reinforcement of the rubber matrix with strong filler-filler bonds and cyclic break down and re-aggregation of softer filler clusters and hence weaker filler-filler bonds. Any type of cluster that is stretched stores energy that is dissipated when the cluster breaks. The free energy density of filler reinforced rubber is described in Equation (23), as the sum of the equilibrium energy density stored in strained rubber matrix ($W_{R}$) and the energy stored in the residual fraction of the softened filler clusters ($W_{A}$) weighted by a factor representatives of the filler volume fraction ϕ:

(23) $$W=\left(1-\phi \right)W_{R}+\phi W_{A}.$$

The DFM’s original form could only describe the material behaviour for uniaxial tension and compression. Lorenz [65,66] suggested a broader generalization of the framework. Juhre and Raghunath [67,68] extended this model further to also include time-dependent effects.

Freund [69] implemented in FEM a generalization of DFM to a fully 3D constitutive model using the concept of representative directions. In the generalized DFM, only the 5th loading cycles were evaluated for the parameters identification (eight material parameters are needed which are identical in all directions, as is required for isotropy).

Li [70] proposed a hybrid between the additive split method and the micromechanical model. The viscoelastic behaviours of the elastomer have been decomposed into a cross-linked elastomer network (incorporating a hyperelastic behaviour) with superimposed free chain network (incorporating a viscous behaviour). Combining the non-affine network model proposed by Davidson [71] with an updated tube model, this law can predict both the loading and unloading response with different strain rates for un-vulcanized and vulcanized rubber with nine physically meaningful constants.

Another physically based model has been introduced by Plagge [72]. The model was validated using non-crystallizing elastomers with the compounds made from EPDM with different amounts of carbon black and curatives. The total energy is the weighted sum of two contributions, as in Equation (23). The free energy density of the strained rubber matrix is described by a non affine tube model. In addition, the concept of hydrodynamic amplification introduces the interaction between filler and polymer. A power law amplification factor is assumed. The hysteresis is introduced via a continuous reforming process (resulting from the break down and reformation of the filler structures inside the rubber).

**Figure 32:**
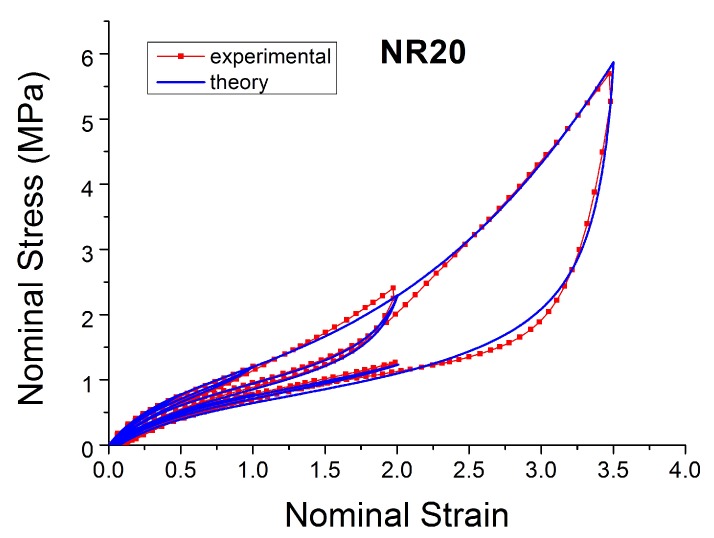
Comparison of the physically based model proposed by Plagge [72] with experimental data for NR20 loaded with cyclic uniaxial stress with different maximum strain amplitude in step up and step down phases (MAXAMP-UPDOWN, Figure 2).

**Figure 33:**
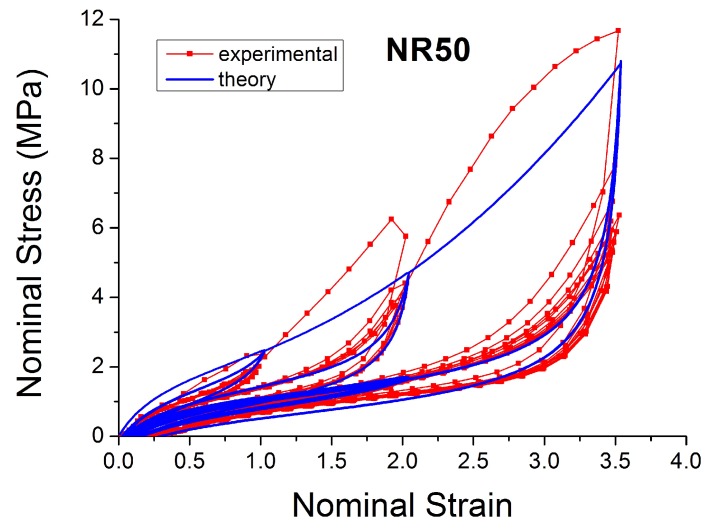
Comparison of the physically based model proposed by Plagge [72] with experimental data for NR50 loaded with cyclic uniaxial stress with different maximum strain amplitude in step up and step down phases (MAXAMP-UPDOWN, Figure 2).

**Figure 34:**
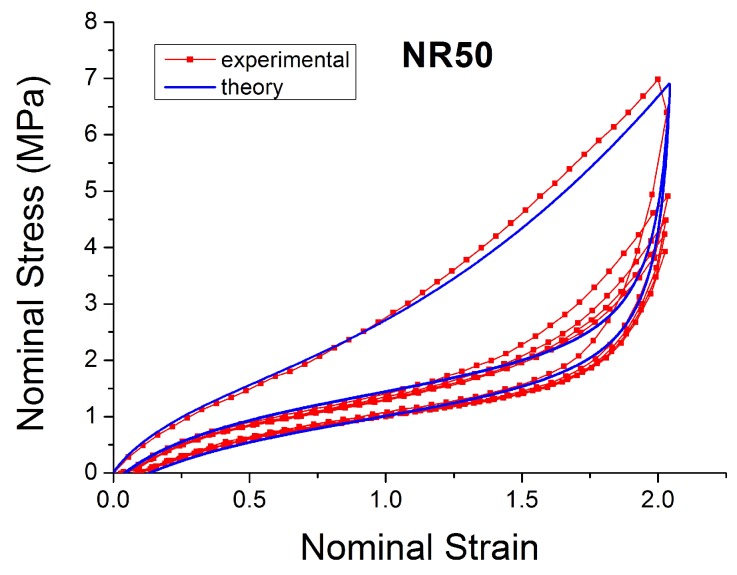
Comparison of the physically based model proposed by Plagge [72] with experimental data for NR50 loaded with cyclic uniaxial test with uniform maximum strain amplitude (MAXAMP-CONST, Figure 1).

If this model is used to fit different compounds such as the Natural Rubber materials tested here, the model shows a good agreement with NR filled with carbon black. As shown in Figure 32 and Figure 33, it is able to reproduce the Mullins effect and all the pre-strain effects, but it fails to reproduce cyclic stress softening. There is not a transition, but the theoretical stress–strain path returns from the steady cycle to the primary, virgin curve, which ignores the cyclic stress softening (Figure 34). The theoretical stress for the maximum strain is the same for the first and following cycles and the stress–strain path is described by the same loading and unloading curves for all the cycles succeeding the initial loading cycle.

In total, Plagge’s model uses seven parameters. Examining the covariance matrix of the variables allowed a statistical analysis to be conducted to study how the parameters are related to each other. The fitting of our compounds under arbitrary deformation histories demonstrates a strong inverse correlation between the two variables defined in the paper [72] as ϕ and $\sigma_{c}$. This is perhaps not surprising as these variables respectively represent the factor that scales elastic and hysteretic stress contributions (thus the amount of hysteresis) and the average critical stress at which rubber-filler structures break down and reform (thus scales the amplitude dependency of the hysteretic stress component).

## 5. Discussion

Table 2 summarises the characteristics of the most widely used and promising models that have been described in this review. The first column lists the name of the model or the first author of the paper where it was introduced, the second column is the year of publication. The third contains information about the number of parameters required to fit the experimental behaviour. The next column shows if the model is predictive. By predictive, we mean a model able to fit the experimental data different from the calibration data. For each kind of nonlinear viscoelastic phenomena, the effectiveness of the models is given. By effectiveness, we mean the ability of reproducing the desired phenomena. In the table, the word *small* means that the model works only for a lower volume fraction of carbon black. As an example, the damage models are unable to model the different loading and subsequent reloading stress–strain path with the consequence that they cannot be expected to predict the cyclic stress softening phenomenon. Essentially, there is a limitation in fitting the experimental behaviour as a consequence of the background theory.

The PRF (rheological model with elastic and viscous components) is the only model that is capable of reproducing the full range of nonlinear viscoelastic effects that are discussed in this paper, but it requires a large number of parameters that are very sensitive to the input test data. In addition, the material model is not predictive in that data fitted to one load case cannot be used to predict a different loading scenario.

The MORPH model shows good fitting for polymers with small amount of filler with a more reasonable number of parameters, but it also becomes less reliable when the amount of filler increases.

Plagge’s model has the advantages that with seven fitting parameters with a specific physical meaning it is able to reproduce some aspects including the influence of the loading history, but it is not able to represent the cyclic stress softening phenomena.

## 6. Conclusions

Over the range of strains and strain rates that rubber based automotive components are expected to operate, the filled vulcanized elastomers display strong nonlinearity, large hysteresis, complex Mullins behaviour, cyclic stress relaxation and permanent set.

No existing constitutive law can model all the nonlinear viscoelastic effects that the filled rubbers exhibit in the regime of interest. In addition, none of these models have a full predictive ability. Hence the realisation of a model to predict all the nonlinear viscoelastic effects still represents a significant challenge for further study.

## Figures and Tables

**Table 1:** Compound formulation; phr (part per hundred of rubber by mass).

-	NR2	NR10	NR20	NR30	NR40	NR50	NR60
**Natural Rubber, SMR CV60**	100	100	100	100	100	100	100
**Carbon Black, FEF N550**	2	10	20	30	40	50	60
**Process oil, 410**	-	1	2	3	4	5	6
**Zinc oxide**	5	5	5	5	5	5	5
**Stearic acid**	2	2	2	2	2	2	2
**Antioxidant/antiozonant, HPPD**	3	3	3	3	3	3	3
**Antiozonant wax**	2	2	2	2	2	2	2
**Sulfur**	1.5	1.5	1.5	1.5	1.5	1.5	1.5
**Accelerator, CBS**	1.5	1.5	1.5	1.5	1.5	1.5	1.5

**Table 2:** Nonlinear viscoelastic models.

Model	Year	Num. Params	Predict.	Hysteresis	Mullins	Pre-Strain	Cyclic Stress Relaxation	Strain Rate
**Ogden [45]**	1999	2 + hyp.			X			
**Dorfmann [32]**	2003	3 + hyp.			X			
**Wrublesky [48]**	2015	3 + hyp.			X			
**Bergstrom-Boyce [51]**	1998	4 + hyp.		X				X
**PRF**	2013	>10		X	X	X	X	X
**MORPH [59]**	2003	8	X	X	small	small	small ($2_{nd}$ cycle)	
**Plagge [72]**	2017	7	X	X		X		X

## Abbreviations

The following abbreviations are used in this manuscript:

NR	Natural Rubber
PHR	Part per Hundred Rubber
SBR	Styrene-Butadiene Rubber
NBR	Nitrile Butadiene Rubber
EPDM	Ethylene Propylene Diene Monomer Rubber
TARRC	Tun Abdul Razak Research Center
SMR	Standard Malaysian Rubber
FEF	Fast Extruding Furnace
HPPD	Hydroxyphenylpyruvate Dioxygenase
CBS	N-Cyclohexyl-2-benzothiazole sulfenamide
DMA	Dynamic Mechanical Analysis
DFM	Dynamic Flocculation Model
PRF	Parallel Rheological Framework
